# 38 Combined ICG and PpIX Fluorescence Imaging of Necrotic and Hypoxic Tissue in Murine Burn Wounds

**DOI:** 10.1093/jbcr/irae036.038

**Published:** 2024-04-17

**Authors:** Aiping Liu, Marien I O Mendoza, Matthew S Reed, Brian Pogue, Angela Gibson

**Affiliations:** University of Wisconsin-Madison, Madison, WI; University of Wisconsin School of Medicine and Public Health, Madison, WI; University of Wisconsin-Madison, Madison, WI; University of Wisconsin School of Medicine and Public Health, Madison, WI; University of Wisconsin-Madison, Madison, WI; University of Wisconsin School of Medicine and Public Health, Madison, WI; University of Wisconsin-Madison, Madison, WI; University of Wisconsin School of Medicine and Public Health, Madison, WI; University of Wisconsin-Madison, Madison, WI; University of Wisconsin School of Medicine and Public Health, Madison, WI

## Abstract

**Introduction:**

Hypoxia is known to play a role in the pathophysiology of burn wound progression and healing. However, the localization of the hypoxic tissue in burns has not been clearly delineated in vivo. Recently, delayed fluorescence signal from Protoporphyrin IX (PpIX), an endogenous fluorescence molecule, has been shown to be a unique reporter of the local oxygen partial pressure in tissue. We have shown that Second-Window Indocyanine Green (SWIG) fluorescence identifies necrotic tissue in burn wounds in both murine models and human subjects. This pilot study aimed to study the feasibility of applying both PpIX and SWIG imaging to identify the necrotic and hypoxic regions of burn wounds, and to determine whether PpIX precursor 5-aminolevulinic acid (5-ALA) enhanced PpIX signals are necessary to identify the hypoxic region of burn early after injury and during healing.

**Methods:**

Nude mice received a full-thickness contact burn on the hind leg with a customized burn device. 24 hours (hr) after ICG administration (@5 mg/kg), two groups of mice (n = 4 per group) with/without 5-ALA injection (@ 250 mg/kg, i.v.) were imaged using an ICG imager and a time-gated PpIX imager at various times post burn. To determine the effect of burn age on fluorescence signals, additional mice were injected with 5-ALA or ICG at 0, 24, or 48 hours post burn (hpb). After imaging, the burned tissues were cryosectioned for microscopic imaging to localize ICG and PpIX. The SWIG signal-to-background ratio (SBR) was calculated as the ratio between the averaged ICG intensity in the burn region and that of its contralateral non-burn region.

**Results:**

SWIG and PpIX signals localized to the burn region with different dynamics. When ICG was administered immediately after burn injury, SWIG SBR was highest (2) at 24 hr after injection and remained elevated (>1.5) three days post injection. When ICG was administered 48 hpb, SWIG SBR 24 hr after injection was less than 1.5. Endogenous PpIX signals in the burns were visible at 24 hpb and peaked 96 hpb. Delayed endogenous PpIX revealed the burn wound was hypoxic 72 hpb. 5-ALA injection at 45 hpb enhanced PpIX signals and allowed the delayed PpIX to reveal hypoxia in the burn wounds at 48 hpb. If injected immediately after burn, 5-ALA enhanced the capability of delayed PpIX to identify hypoxia regions as early as 6 hpb. Microscopically, co-localization of SWIG and PpIX signals was confirmed in the burn wounds.

**Conclusions:**

The pilot study has demonstrated the potential of a novel combined ICG and PpIX imaging to identify the necrotic and hypoxic tissues in burn wounds in vivo. Like ICG, we show that endogenous PpIX is sufficient to identify the burn region as early as 24 hpb and to identify hypoxia in the healing phase. 5-ALA enhanced delayed PpIX signals to reveal hypoxia in burns in the early injury phase.

**Applicability of Research to Practice:**

Novel non-contact methods to identify necrotic and hypoxic regions in burn wounds in vivo may enhance our understanding of burn wound progression and healing.

